# Non-Monotonic Relation between Noise Exposure Severity and Neuronal Hyperactivity in the Auditory Midbrain

**DOI:** 10.3389/fneur.2016.00133

**Published:** 2016-08-25

**Authors:** Lara Li Hesse, Warren Bakay, Hui-Ching Ong, Lucy Anderson, Jonathan Ashmore, David McAlpine, Jennifer Linden, Roland Schaette

**Affiliations:** ^1^UCL Ear Institute, London, UK; ^2^Klinik für HNO, Universitätsklinikum Schleswig-Holstein, Lübeck, Germany; ^3^Department of Neuroscience, Physiology and Pharmacology, University College London, London, UK

**Keywords:** tinnitus, hearing loss, mouse model, computational model, noise exposure, neuronal hyperactivity, cochlear damage, hidden hearing loss

## Abstract

The occurrence of tinnitus can be linked to hearing loss in the majority of cases, but there is nevertheless a large degree of unexplained heterogeneity in the relation between hearing loss and tinnitus. Part of the problem might be that hearing loss is usually quantified in terms of increased hearing thresholds, which only provides limited information about the underlying cochlear damage. Moreover, noise exposure that does not cause hearing threshold loss can still lead to “hidden hearing loss” (HHL), i.e., functional deafferentation of auditory nerve fibers (ANFs) through loss of synaptic ribbons in inner hair cells. While it is known that increased hearing thresholds can trigger increases in spontaneous neural activity in the central auditory system, i.e., a putative neural correlate of tinnitus, the central effects of HHL have not yet been investigated. Here, we exposed mice to octave-band noise at 100 and 105 dB SPL to generate HHL and permanent increases of hearing thresholds, respectively. Deafferentation of ANFs was confirmed through measurement of auditory brainstem responses and cochlear immunohistochemistry. Acute extracellular recordings from the auditory midbrain (inferior colliculus) demonstrated increases in spontaneous neuronal activity (a putative neural correlate of tinnitus) in both groups. Surprisingly, the increase in spontaneous activity was most pronounced in the mice with HHL, suggesting that the relation between hearing loss and neuronal hyperactivity might be more complex than currently understood. Our computational model indicated that these differences in neuronal hyperactivity could arise from different degrees of deafferentation of low-threshold ANFs in the two exposure groups. Our results demonstrate that HHL is sufficient to induce changes in central auditory processing, and they also indicate a non-monotonic relationship between cochlear damage and neuronal hyperactivity, suggesting an explanation for why tinnitus might occur without obvious hearing loss and conversely why hearing loss does not always lead to tinnitus.

## Introduction

Epidemiological data suggest a close relation between hearing loss and tinnitus. For example, most tinnitus patients also have a certain degree of hearing loss (1, 2), tinnitus prevalence rises with hearing loss (3), 75–90% of patients with otosclerosis experience tinnitus (4, 5), as do 80% of patients with idiopathic sudden sensorineural hearing loss (6). However, upon closer inspection, the relation between hearing loss and tinnitus appears quite heterogeneous. For example, even though there is a general trend for the tinnitus pitch to coincide with frequencies affected by hearing loss (7), clear correlations between the shape of the hearing threshold curve in the audiogram and tinnitus characteristics have only been observed for homogeneous study groups like noise-exposed workers (8), but not for general tinnitus patient samples (9, 10). Moreover, a significant fraction of individuals with tinnitus show no obvious signs of hearing loss (11, 12), and conversely many hearing-impaired listeners do not experience tinnitus (13).

Clinically, hearing loss is quantified through pure-tone audiometry, which measures thresholds for detecting tones in quiet. However, hearing thresholds alone only convey a limited picture of actual cochlear damage. The degree of outer hair cell loss, for example, only shows a moderate correlation to hearing threshold shifts (14), and cochlear dead regions, i.e., cochlear regions with severe loss of inner hair cells (IHCs), cannot be reliably detected through audiometry alone (15). Moreover, hearing thresholds are poor predictors of listening performance (16), and some hearing problems might not be detected by audiometry at all.

Thus, hearing thresholds do not provide a complete picture of hearing function. Indeed, recent evidence even suggests a considerable degree of “hidden hearing loss” (HHL) might be present despite a normal audiogram. Investigations in mice have shown that noise exposures producing only a temporary elevation of hearing thresholds can lead to loss of synaptic ribbons at synapses between IHCs and auditory nerve fibers (ANFs), permanently reducing the amplitude of wave I of the auditory brainstem response (ABR) (17). ANFs with high response thresholds appear particularly vulnerable to this kind of deafferentation, whereas fibers with low thresholds seem to be more resilient (18). Moreover, HHL might also develop through age-related processes in humans (19) as well as mice (20). Significant reductions of ABR wave I have also been reported for tinnitus subjects with clinically normal audiograms (21, 22), suggesting that HHL might not only cause a degradation of the auditory nerve response but also affect auditory processing beyond the ear.

Peripheral hearing loss can lead to pronounced changes in the central auditory system, with reduced inhibitory neurotransmission (23–27), increased excitatory neurotransmission or neuronal excitability (23, 25, 27, 28), and changes reported in gene expression (29). Moreover, spontaneous neuronal activity in the auditory system is altered in a non-intuitive fashion after hearing loss. While ANFs generally show reduced (30) or unchanged (31) spontaneous activity following induction of various kinds of cochlear damage, spontaneous firing rates in the central auditory system are reported to increase. Cochlear damage through exposure to noise or ototoxic drugs generates elevated spontaneous firing rates in the dorsal (32–34) and ventral (35) cochlear nuclei, the inferior colliculus (36–38), and the auditory cortex (39, 40). This neuronal hyperactivity has been linked to behavioral markers of tinnitus (34, 41–43). Moreover, the level of hyperactivity was proportional to the degree of hearing loss or cochlear damage (33, 36, 44).

Here, we investigate how noise-induced HHL affects spontaneous and evoked neuronal activity in the auditory midbrain of mice. In contrast to most previous studies employing unilateral noise exposure, we employed bilateral noise exposure – more typical of the noise exposure generally encountered by human listeners. After exposing two groups of mice to different levels of noise (100 dB SPL to induce HHL, 105 dB SPL to induce permanent changes in hearing thresholds), we compared the effects of “hidden” and “obvious” hearing loss on IC responses. Although spontaneous firing rates increased in both exposure groups, the increase was greatest in the animals exposed to the lower (100 dB SPL) sound level, which showed ANF deafferentation without permanent shifts in hearing thresholds. Using a computational model, we demonstrate that this non-monotonic relationship between the degree of cochlear damage and the degree of spontaneous neuronal hyperactivity is explicable in terms of different degrees of deafferentation of high- and low-threshold ANFs, suggesting that specific ANF deafferentation patterns could differ significantly in their effect on auditory function. Our results thus indicate that differences in the specific patterns of cochlear damage, which cannot be reliably determinated from hearing threshold measurements alone, might account for some of the heterogeneity observed in the relation between hearing loss and tinnitus.

## Materials and Methods

### Subjects

Subjects were 23 male CBA/Ca mice. Mice were 7–19 weeks old at the time of noise exposure. Control animals were age-matched littermates. ABRs were recorded 1–14 days prior to, 1 day after, and 4 weeks after noise exposure. IC recordings took place 4 weeks after noise exposure. At the end of the final experiment, mice were overdosed with an intra-peritoneal (i.p.) injection of sodium pentobarbital. All experiments were performed in accordance with the United Kingdom Animal (Scientific Procedures) Act of 1986.

### Noise Exposure

Mice were anesthetized with ketamine and medetomidine (i.p.) and positioned in a custom-made sound-proof booth on a heated pad directly underneath the center of a speaker (Stage Line PA Horn Tweeter MHD-220N/RD) positioned 45 cm above. The speaker was calibrated prior to each use to ensure that the frequency response was flat (±2 dB) over the 8–16 kHz range. Noise exposure was performed with an octave-band noise (8–16 kHz) at 100 or 105 dB SPL for 2 h. Noise stimuli were generated using an RX6 processor (Tucker-Davis Technologies, TDT), attenuated as required (TDT PA5) and amplified (TDT SA2). During the noise exposure, pedal reflex and breathing rate were checked every 30 min. Control animals were not exposed to any additional sound beyond the normal background noise in the animal unit.

### Auditory Brainstem Response Recording and Analysis

For ABR recordings, animals were anesthetized with an i.p. injection of ketamine and medetomidine. ABR recordings were obtained using subdermal needle electrodes (Rochester Medical), one inserted at the vertex, and one each behind the ipsilateral and contralateral pinnae. Electrode signals were low-pass filtered (7.5 kHz cut-off frequency, 12 dB per octave) and recorded at 24 kHz sampling rate (TDT RA4LI, RA4PA and RX5). For analysis, ABR data were filtered using a bandpass filter (100–3000 Hz, 5th-order Butterworth filter). Stimuli were either tone pips (5 ms total duration with 1.5 ms rise/fall time; frequencies 6, 8, 11, 16, 24, 32, and 48 kHz; intensities 0–80 dB SPL in 5 dB steps) or clicks (50 μs duration, 0–80 dB SPL in 5 dB steps), both delivered at a rate of 20/s. Stimuli were generated using a TDT RX6 processor, attenuated as needed (TDT PA5), amplified (TDT SA2), and presented in free-field condition with the speaker (TDT FF1) positioned at a 45° to the animal’s axis at a distance of approximately 15 cm. The ear contralateral to the speaker was blocked using a foam earplug. Before the start of each experiment, the transfer function of the speaker was measured with a microphone (4939, Brüel and Kjaer) placed at the location of the animal’s ear with the animal in place. This function was used to calibrate individual tones so that the overall output of the speaker was flat across frequency to ±3 dB. ABR thresholds were determined visually by estimating the lowest sound level at which deflections in the ABR waveform were judged to be greater than the background variability in the waveforms. Measurements of wave amplitudes were performed using custom Matlab software: a time window containing the wave of interest was selected by the user, and the software then detected maxima and minima of the ABR traces within that window. ABR wave amplitudes were measured from the peak to the following trough.

### Extracellular Recordings in Inferior Colliculus

Animals were anesthetized with an i.p. injection of ketamine and medetomidine, followed by administration of dexamethasone and atropine sulfate. Lactated Ringers solution was given every 2 h to maintain hydration. The animal’s temperature was maintained at 37.5°C using a homeothermic blanket connected to a rectal thermistor. Breathing rate was monitored throughout the surgery, and then at 45-min intervals throughout the recording. Once the pedal reflex had been abolished, the mouse was placed in a nose clamp to stabilize the head while leaving the ears free. To access the IC, a large craniotomy (≈4.5–6.5 mm posterior to Bregma, 0.5–3.5 mm lateral to midline) was performed on the right-hand side, revealing the full surface of the right IC. Extracellular, multi-unit recordings were made using single-shank silicon multi-electrodes with 16 recording sites (1 × 16 linear array with 100 μm spacing; NeuroNexus). This arrangement of electrode sites enabled sampling across the full extent of the central nucleus of the IC. The probe was advanced manually until the tip just touched the collicular surface. Using a remote hydraulic microdrive (Neurocraft, FHC Inc.), the electrode array was then advanced rapidly by 2000 μm, to minimize the duration of tissue compression during the initial penetration, and then retracted by 500 μm. Electrode signals were recorded at 24 kHz sampling rate and bandpass-filtered between 300 and 9000 Hz (TDT RX5).

To record frequency-response areas (FRAs), tone pips were played in sequential order from 4 to 70 kHz in one-eighth octave steps, with sound level ranging from 0 to 80 dB SPL in 5 dB steps. Tone pip duration was 100 ms, with a rise/fall time of 5 ms, and the inter-stimulus-interval was 400 ms. The speaker was positioned at a 45° to the animal’s axis and at a distance of approximately 15 cm from the animal’s left ear. The right ear was plugged with a sound-attenuating plug. The equipment for sound generation and delivery was the same as for the ABR measurements. The FRA measurement was repeated three times.

To obtain a measure of the spontaneous firing rates, we recorded 100 epochs of 500 ms duration without sound presentation. The speaker was unplugged during the recording of spontaneous activity.

### Analysis of IC Data

To obtain multiunit activity, the electrode signals were first stripped of the local field potential by applying an additional high-pass filter with cut-off at 600 Hz, and action potentials were then classified using a latent variable spike-sorting algorithm (45) to separate multi-unit clusters from background noise. Characteristic frequencies (CFs) and thresholds of the multiunits were determined visually from the FRAs. Multi-unit recordings were considered to originate from the central nucleus of the IC (ICc) when there was a clear progression of CFs along the length of the linear electrode array. Recordings from electrodes that deviated from this CF progression were excluded from further analysis. Rate-level functions at CF were then derived from the FRA data. Spontaneous firing rates were determined by calculating the average firing rate over the 100 repetitions of the silent epoch of 500 ms duration.

### Cochlear Immunohistochemistry for Synaptic Ribbon Counts

After the end of an IC recording, the cochleae were harvested and fixed in 4% paraformaldehyde. For immunohistochemistry, the organ of Corti was left in the temporal bone in position with no decalcification in order to identify the precise location of the cells in each cochlea. The tissue was accessed by removing the overlying apical bony covering and the tectorial membrane removed using micropipette aspiration or fine forceps. The procedure therefore differs from more conventional histological procedures and is a novel approach. To identify the synaptic structures after tissue permeabilization, we employed mouse anti-CtBP2 (#612044, BD Transduction Laboratories), used at 1:200, or simultaneously with rabbit anti-GluR2/3 (#AB1506, Millipore Bioscience), used at 1:50, incubated at 4°C overnight with a standard lysine block. The ribbon protein CtBP2 was then labeled by secondary antibodies conjugated to ATTO425 (TEFLabs); the postsynaptic terminals were labeled using biotinylated goat anti-rabbit IgG (#BA-1000, Vector) and subsequent incubation with either fluorescein or Alexa 488 conjugated to streptavidin (#SA-5001 Vector). The temporal bone was mounted for viewing hair cells from scala media and images were acquired as z-stacks with step sizes of 0.2–0.5 μm with a 63 × 1.0 NA objective. For such *in situ* identifications, multiphoton imaging was used in an upright LSM 510 confocal microscope (Zeiss). Ribbons were identified from z-projections and only those with both labels positively identified and were manually counted. Ribbons per cell were typically determined as averages from the groups of 8–12 IHCs from the 10–30 kHz region of the cochleae. No account was taken individual variations in the cochlear frequency place map between animals. The differences between the resulting data and reported figures from decalcified and dissected tissue [e.g., Ref. (17)] may reflect differences in the sampling processes.

### Computational Model

We have set up a computational model of the early auditory system, as described below, to study the effects of different patterns of ANF deafferentation on spontaneous activity in the central auditory system. The model is based on previous models of the effects of loss of inner and outer hair cells and damage to hair cell stereocilia (46, 47) as well as selective deafferentation of high-threshold ANFs (21).

### Auditory Nerve Response Stage of the Model

Auditory nerve responses are modeled with a rate-level function that represents the average response of a small population of ANFs, comprising low-, medium-, and high-threshold fibers, with similar CFs (46). The effects of different types of cochlear damage on AN responses are then captured by adjusting the shape of the ANF population rate–intensity function. Selective loss of high-threshold ANFs is captured by scaling down responses to high sound intensities and reducing the maximum firing rate of the ANF population. The resulting rate–intensity function f*(*I*) is then

$$f^{*}(I)=f(I)-d_{h}{\left[f(I)-f_{h}\right]}_{+}$$

where []_+_ denotes positive rectification, 0 ≤ *d_h_* < 1 represents the fraction of missing high-threshold fibers, and *f_h_* = 100 spikes/s is the firing rate where deafferentation starts to influence the AN population response. In addition to selective deafferentation of high-threshold ANFs, we also consider cases where all fiber types get deafferented:

$$f^{*}\left(I\right)=\left(f\left(I\right)-d_{h}{\left[f\left(I\right)-f_{h}\right]}_{+}\right)d_{a}$$

The parameter *d_a_* scales the whole AN population rate-level function, and thus (for *d_h_* = 1) models equal deafferentation of all fiber types when it is set to a value between 0 and 1. When both *d_h_* and *d_a_* are set to values < 1, deafferentation with a bias toward high-threshold fibers is created. Examples of the resulting AN population rate-level functions are shown in Figure 4A. To model hearing threshold increase, we increase the response threshold without changing spontaneous and maximum firing rate, based on the model for outer hair cell loss presented in Ref. (46, 47). Examples of rate–intensity functions with threshold increase and ANF deafferentation can be seen in Figure 4B.

### Central Auditory System Stage of the Model

The central auditory system stage comprises a neuronal circuit where projection neurons (PNs) receive inhibition from two different types of inhibitory interneurons, one of them providing wide- and the other narrow-band inhibition. This circuit architecture is based on the circuitry of the dorsal cochlear nucleus (DCN) [reviewed in Ref. (48)], as this circuit is probably the best-characterized circuit in the auditory brainstem. However, by adjusting the strength of inhibition (governed by the two parameters *g_w_* and *g_n_*), a variety of different response types can be generated [see Ref. (47), for more details]. In this study, we used *g_w_* = 0.5 and *g_n_* = 0.6, which yields model PNs with monotonous rate-level functions, as typically observed in the inferior colliculus. Moreover, for the application of the model to data from the IC, we have now also included a response threshold for the PN as an additional parameter to account for the fact that spontaneous firing rates in the IC are generally quite low, and especially lower than in the cochlear nucleus (36, 48, 49). The firing rate response *r* of the PN to excitatory input at rate *f* (provided here through the activity of ANFs) and input from the inhibitory interneurons at rates *w* and *n* is then given by

$$r=R\left(f,w,n\right)=r_{\text{max}}\text{tanh}\left({\left[f-g_{w}w-g_{n}n-\text{th}\right]}_{+}\text{​}/r_{\text{max}}\right)$$

where *r*_max_ = 300 spikes/s is the maximum firing rate of the PN, and *th* = 40 spikes/s is the response threshold.

In the model, we assume that the mean firing rate of the PNs is stabilized at a certain target level (i.e., the mean activity obtained for input from an undamaged cochlea) through homeostatic plasticity, which scales the strength of excitatory and inhibitory synapses onto the PN in opposite directions. When the mean activity of the PN is permanently below the target, homeostatic plasticity increases excitation and decreases inhibition to bring activity back up to the target level. Prolonged increases in activity lead to changes in the opposite direction. Synaptic scaling is implemented through the homeostasis factor *h* (limited to values between 1/3 and 3 to account for physiological constraints on synaptic strength):

$$r=R\left(f,w,n,h\right)=r_{\text{max}}\text{tanh}\left({\left[hf-\frac{g_{w}}{h}w-\frac{g_{n}}{h}n-\text{th}\right]}_{+}\text{​}/r_{\text{max}}\right)$$

The exact value of *h* required to reach the desired target mean activity for a certain pattern of cochlear damage is determined numerically.

All data analysis as well as implementation and evaluation of the model were done using MATLAB (The MathWorks Inc., Natick, MA, USA). To test for significant differences, *t* tests were used unless otherwise stated.

## Results

We investigated the effects of two different levels of noise exposure – 100 dB SPL and 105 dB SPL – on young adult male CBA/Ca mice, using ABR measurements, cochlear immunohistochemistry, and multiunit recordings from the IC. Noise exposure was performed with an octave-band noise (8–16 kHz) at 100 dB SPL (*n* = 6 animals) or 105 dB SPL (*n* = 10 animals) for 2 h, while the animals were anesthetized. The control group consisted of eight age-matched mice.

### The Effect of Noise Exposure on ABR Responses

**Figure 1:**
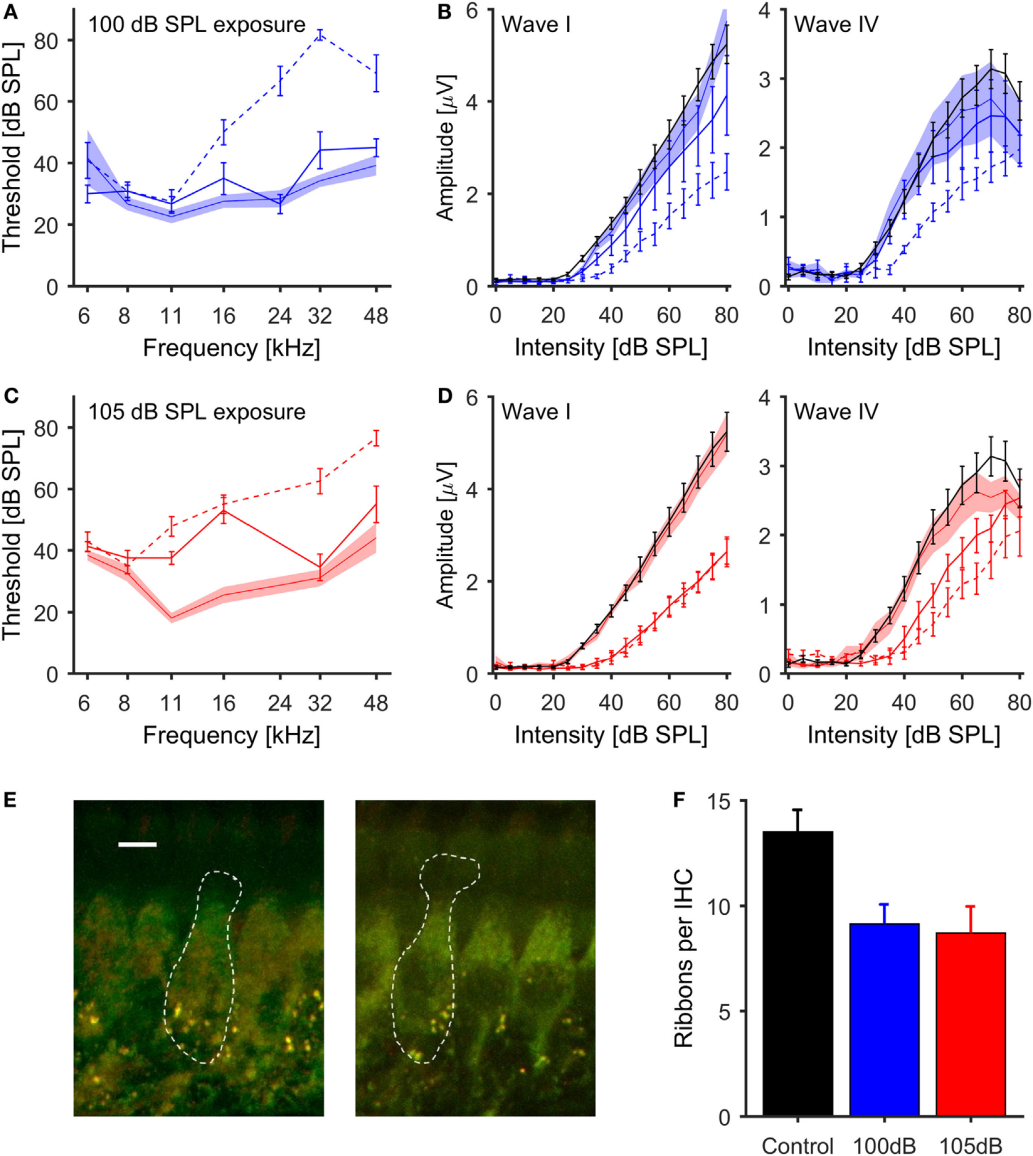
**Effects of noise exposure on the auditory periphery of mice (A,C)**. ABR thresholds for tone-pip stimulation. Pre-exposure thresholds ±1 SEM are indicated by the shaded areas, thresholds 1 day postexposure by dashed lines, and 4 weeks postexposure by solid lines [**(A)** – 100 dB SPL exposure, blue; **(C)** – 105 dB SPL exposure, red]. **(B,D)** Growth function of the amplitudes of ABR waves I and IV (50 μs clicks) for control (black lines) and noise-exposed mice [**(B)** – 100 dB SPL exposure in blue; **(D)** – 105 dB SPL exposure in red]. Pre-exposure data ± 1 SEM are indicated by the shaded areas, 1 day postexposure by dashed and 4 weeks postexposure by solid lines. Both levels of noise exposure caused a significant reduction of ABR wave I amplitude. **(E)** Examples of inner hair cells from a control mouse (left, scale bar = 5μm) and a mouse exposed to 105 dB SPL noise (right), with synaptic ribbons labeled green (anti-CtBP2) and postsynaptic terminals red (anti-GluR2/3). **(F)** Mean number of synaptic ribbons per IHC; black, control (*n* = 6 cochleas); blue, 100 dB SPL exposure (*n* = 3 cochleas); red, 105 dB SPL (*n* = 4 cochleas). There was a significant difference in the ribbon count per IHC between control and noise exposed ears for both noise conditions (ANOVA, *p* = 0.03).

Auditory brainstem responses were measured before, 1 day after, and 4 weeks following noise exposure. At 1 day postexposure, both exposure groups exhibited a shift in response thresholds for tone pips of around 20–40 dB at frequencies above 11 (105-dB-SPL exposure, Figure 1C) or 16 kHz (100-dB-SPL exposure, Figure 1A). Four weeks after noise exposure, ABR thresholds had recovered in the 100-dB-SPL exposure group, such that they were no longer significantly different from the pre-exposure values at all test frequencies (all *p*-values > 0.1). In the 105-dB-SPL group, thresholds remained significantly elevated by around 20–25 dB at 11 and 16 kHz (*p* < 0.01 at both frequencies), but recovered, and were no longer significantly different from pre-exposure levels, at 32 and 48 kHz (*p* > 0.1 at both frequencies, Figure 1C).

The effects of noise exposure on the ABR were further investigated by analyzing the amplitudes of ABR waves I and IV in response to broadband clicks. Four weeks after noise exposure, both exposure groups displayed significantly lower amplitudes of click-evoked ABR wave I compared to control mice (*p* < 0.01, repeated measures ANOVA). The strongest reduction in ABR amplitude, in conjunction with an increase of the response threshold, was observed in the group exposed to 105-dB-SPL noise (Figure 1D). These changes in the magnitude of ABR wave I are indicative of deafferentation of ANFs (17, 18, 50).

In contrast to the effects of noise exposure on wave I of the ABR, amplitudes of wave IV in the 100-dB-SPL exposure group were virtually identical to those in control animals for click intensities up to 45 dB SPL, and then showed a slightly shallower growth at higher intensities (Figure 1B, right panel). In contrast, in the 105-dB-SPL group, the average amplitudes of ABR wave IV were reduced at low and medium sound intensities, but reached those of control animals at high sound intensities, even though the threshold was shifted by about 15 dB, similar to the elevation in wave I thresholds for this group. Moreover, the slope of the amplitude growth function above threshold was very similar to that of the control group (Figure 1D, right panel).

### The Effect of Noise Exposure on Inner Hair Cell Synapses

To confirm that the observed reduction of ABR wave I amplitude was related to deafferentation of AN fibers, cochleae were extracted from representative animals from the control (*n* = 6), the 100-dB-SPL (*n* = 3) and the 105-dB-SPL (*n* = 4) groups, and loss of synaptic ribbons was quantified using immunohistochemistry (see Materials and Methods). Representative examples from a control mouse and a mouse exposed at 105 dB SPL are shown in Figure 1E. Ribbon counting was carried out in up to three regions of the cochlea for each animal, and the counts were averaged across these regions. Compared to control animals, both the 100- and 105-dB-SPL group showed a reduced number of synaptic ribbons per IHC (Figure 1F). There was a significant effect of noise exposure on the ribbon count (*p* = 0.03, ANOVA).

### Elevated Spontaneous Neural Activity Following Noise Exposure

**Figure 2:**
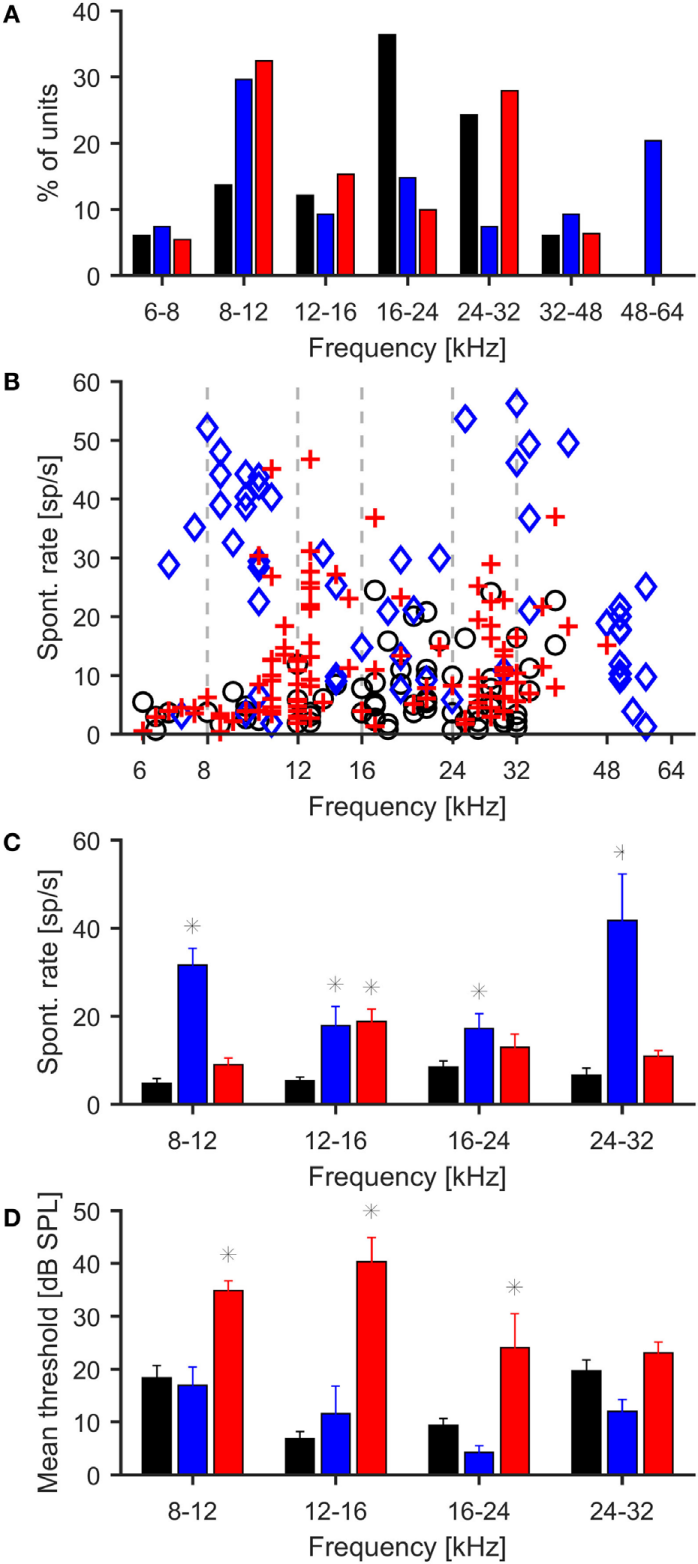
**Effects of noise exposure on neuronal responses in the inferior colliculus**. **(A)** Distribution of characteristic frequencies of multi-units for control (black bars) and mice exposed to 100 (blue bars) and 105 dB SPL noise (red bars). In control mice, most units are found in the range from 16 to 24 kHz, whereas in both groups of exposed mice, CFs in the range from 8 to 12 kHz are most frequently encountered. **(B)** Spontaneous firing rates of individual IC multi-units, black = control, blue = 100-dB-SPL exposure, red = 105-dB-SPL exposure. **(C)** Spontaneous firing rates of multi-unit recordings grouped by characteristic frequencies. Asterisks denote significant differences from control (*p* < 0.01, *t* test). Only frequency ranges with at least five recordings from the control mice have been included in this analysis. **(D)** Average response thresholds of IC recordings, conventions as in plots above. Thresholds were significantly increased in the 105 dB exposure group for 8–12, 12–16, and 16–24 kHz.

In addition to measuring wave IV of the ABR, we also assessed the effects of noise exposure on central auditory function by making multi-unit recordings in the central nucleus of the right IC (ICc) of (anesthetized) noise-exposed and control mice using 16-channel single-shank electrode arrays (see Materials and Methods). A total of 66 multi-unit recordings were obtained from control animals, 54 recordings from animals exposed at 100 dB SPL, and 111 recordings from animals exposed at 105 dB SPL. In control animals, the largest fraction of multi-unit recordings had CFs in the range of 16–24 kHz, commensurate with the most sensitive region of the mouse audiogram. However, in both noise exposure groups, CFs in the range of 8–12 kHz, i.e., near the low-frequency end of the octave-band noise used for the acoustic over-exposure, were most frequently encountered (Figure 2A). Similar results were obtained for best frequencies, i.e., the tone frequencies evoking maximal firing rates (not shown).

In control animals, CF-tone evoked thresholds of ICc recordings ranged from 0 to 45 dB SPL, with a mean of 15.7 ± 1.3 dB SPL. The range of thresholds recorded from the ICc of noise-exposed mice was similar (0–60 dB SPL for 100-dB-SPL exposure and 0–65 dB SPL for 105-dB-SPL exposure); however, the mean threshold of 31.6 ± 1.4 dB SPL in the 105 dB SPL group was significantly higher than in the control group (*p* < 0.01, Figure 2D), corresponding to the threshold shift that was also observed in the ABR recordings. The mean threshold for recordings from the 100 dB SPL exposure group was 17.4 ± 1.7 dB SPL, which did not differ significantly from control.

Spontaneous firing rates were generally low in the control animals at 7.0 ± 1.3 spikes/s. In both exposure groups, the average spontaneous firing rates were significantly increased compared to control animals (*p* < 0.01 in both cases). The average spontaneous firing rate was 26.3 ± 4.2 spikes/s in the 100-dB-SPL exposure group and 11.7 ± 3.1 spikes/s in the 105-dB-SPL exposure group. The difference in spontaneous firing rate between the two noise exposure groups was also significant (*p* < 0.01). To test for significant differences in spontaneous firing rates in different frequency regions, recordings were grouped according to their CFs (8–12 kHz, 12–16 kHz, 16–24 kHz, 24–32 kHz). For CFs <8 kHz and >32 kHz, there were too few recordings in the control data to allow for meaningful statistical analysis. In the control group, average spontaneous rates were around 6–8 spikes/s across frequencies. Spontaneous firing rates in the 100-dB-SPL group were significantly increased in recordings with CFs from 8 to 12 kHz, 12 to 16 kHz, and 16 to 24 kHz (*p* < 0.01 in all cases). In the 105-dB-SPL group, a significant increase in spontaneous firing rates was seen in the 12–16 kHz range (*p* < 0.01). Spontaneous firing rates were also higher than in control mice in the 8–12, 16–24, and 24–32 kHz frequency ranges, but the increases did not achieve significance.

**Figure 3:**
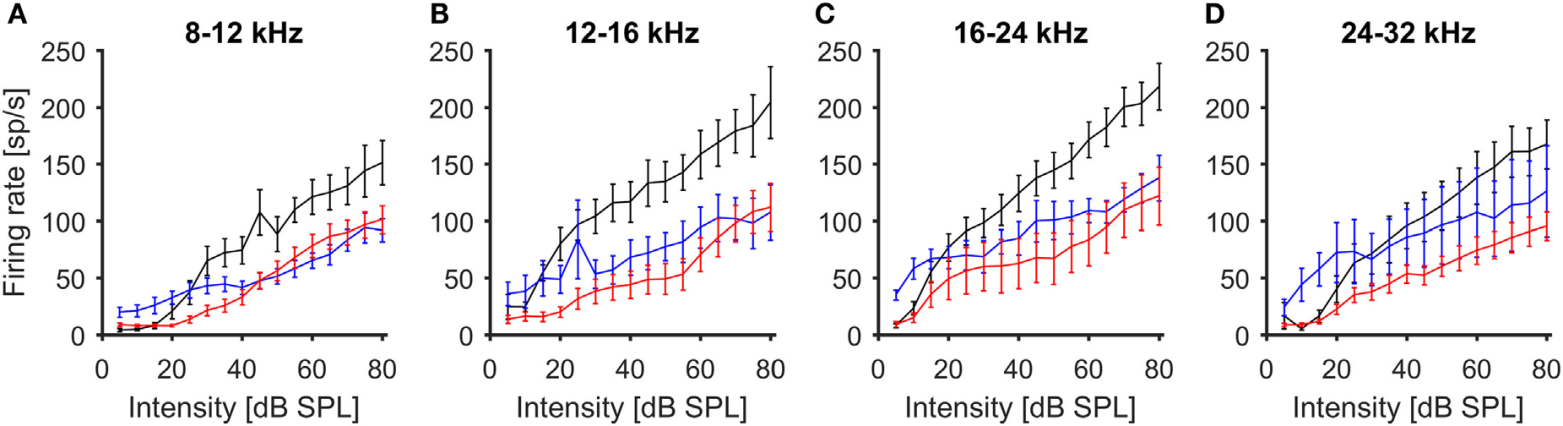
**Average rate-level functions of IC units in response to stimulation with 100 ms tone pips (mean ± 1 SEM)**. Multi-unit recordings have been grouped by CF [**(A)** 8–12 kHz, **(B)** 12–16 kHz, **(C)** 16–24 kHz, **(D)** 24–32 kHz]. Black – control, blue 100-dB-SPL exposure, red 105-dB-SPL exposure.

To further characterize the effects of noise exposure on the response properties of ICc recordings, we also analyzed rate-vs.-intensity functions for responses to CF tones. For this purpose, the multi-unit recordings were grouped according to their CFs, using the same frequency ranges as for the analysis of spontaneous firing rates. The average rate-vs.-intensity functions for the control group showed a monotonic response growth with increasing stimulus intensity, reaching discharge rates of 150–200 spikes/s at 80 dB SPL (Figure 3). In both exposure groups, on the other hand, the slopes of the rate-vs.-intensity functions were shallower on average, and the maximum responses were reduced compared to those in control animals (Figure 3). However, recordings from the 100-dB-SPL group showed elevated spike rates at sound intensities close to threshold, consistent with greater spontaneous activity.

### Accounting for the Non-Monotonic Effect of Noise Exposure on Spontaneous Neural Activity

Perhaps the most surprising outcome of the ICc recording experiments was that noise exposure at 100 dB SPL resulted in a greater increase in spontaneous firing rates than did the 105-dB-SPL exposure. This was despite the fact that 100 dB SPL exposure generated less hearing loss (Figure 1A) and had less effect on the magnitude of ABR wave I (Figure 1B). Our expectation had been that animals in the 105-dB-SPL exposure group would show the greatest increase in spontaneous firing rates, since earlier studies had reported a proportionality between the degree of cochlear damage or threshold increase and the magnitude of spontaneous neuronal hyperactivity [e.g., Ref. (33, 36)], which we had also observed in our earlier modeling results (21, 46, 47, 51).

However, our most recent modeling study has suggested that whereas deafferentation of high-threshold ANFs (which show little or no spontaneous activity) leads to neuronal hyperactivity in the central auditory system, deafferentation of low-threshold fibers (with high spontaneous activity) could even reduce spontaneous firing rates (52). Moreover, while high-threshold ANFs appear to be most susceptible to noise-induced deafferentation (18), reductions in the amplitude of ABR wave I might arise when only low-threshold ANFs are deafferented; recent data suggest that the contribution of high-threshold fibers to the amplitude of ABR wave I is negligible (50). Since mice from the 105-dB-SPL group showed a greater reduction in ABR wave I amplitude with a shallower slope of the amplitude growth function than the mice exposed to noise at 100 dB SPL (Figure 1B), we hypothesized that the 105-dB-SPL-exposed mice might have a higher degree of deafferentation of low-threshold fibers than the 100-dB-SPL mice. We therefore employed the model to explore whether this hypothesis might account for the observed non-monotonic relationship between cochlear damage and IC hyperactivity.

**Figure 4:**
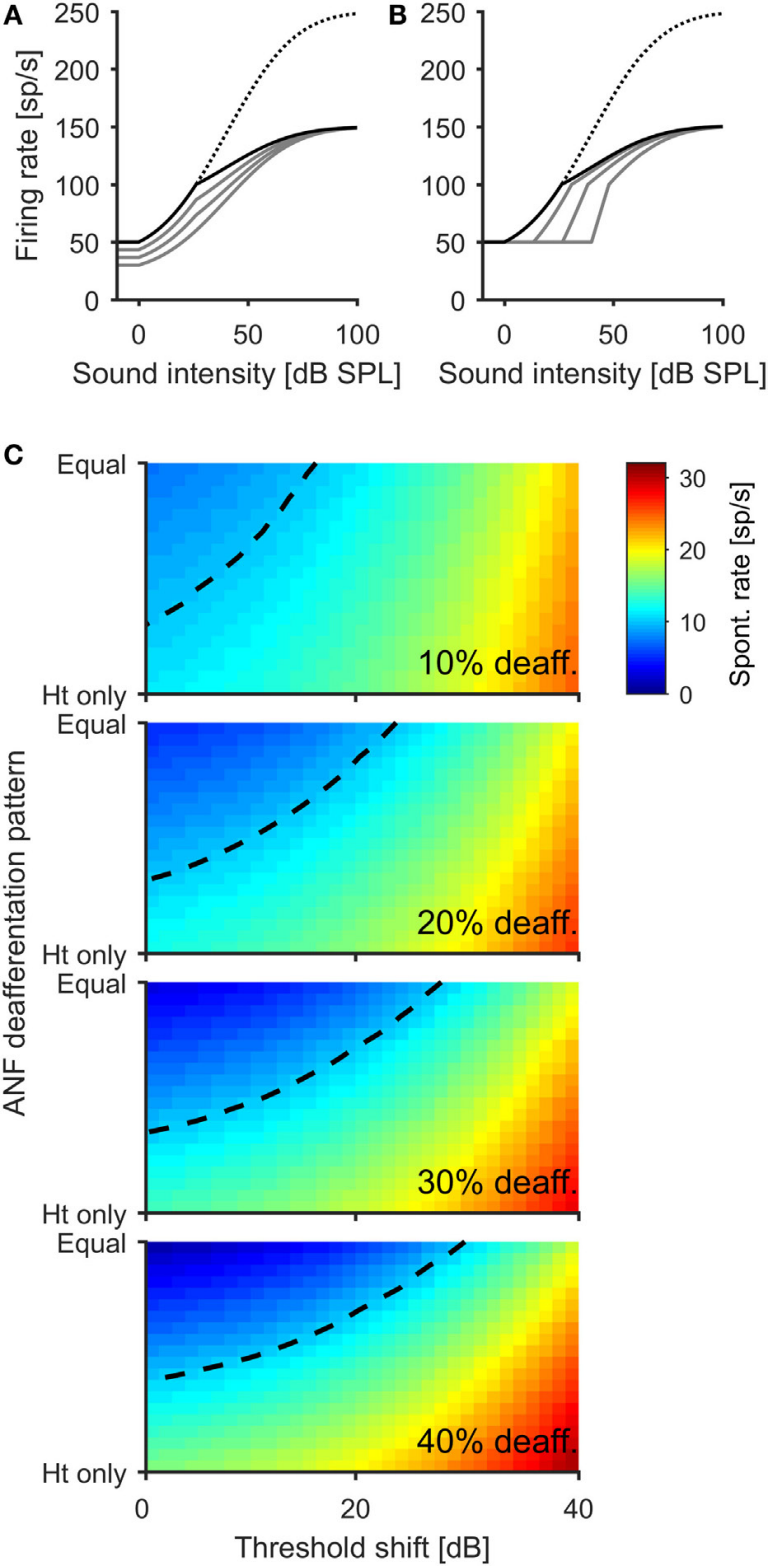
**A computational model offers an explanation for the non-linear relation between the degree of cochlear damage and spontaneous firing rates in the IC**. **(A)** Illustration of how different ANF deafferentation patterns is modeled for an overall deafferentation 40% of ANFs. The dotted line indicates normal AN population rate–intensity function with no deafferentation, and the solid black line depicts the AN population rate–intensity function for a scenario in which only high-threshold fibers become deafferented. The gray lines show the transition with increasing deafferentation of low-threshold fibers, until all fibers are affected to the same degree (lowest gray line). **(B)** Modeling the effect of additional hearing threshold increase. The black line depicts the AN population response function for the scenario with 40% AN fiber deafferentation affecting only high-threshold fibers. The gray lines show the effects of 13.3, 26.7, and 40 dB threshold increase through damage to outer hair cells. **(C)** Color coded depiction of the spontaneous firing rate of a principal neuron in the central auditory system after activity stabilization through homeostatic plasticity in dependence upon ANF deafferentation pattern and threshold increase. Four different degrees of overall ANF deafferentation were modeled, from 10 (top panel) to 40% (bottom panel). The dashed line indicates the normal, healthy level of spontaneous activity. While both deafferentation of high-threshold fibers and threshold increase lead to an increase of spontaneous activity of the model neuron, deafferentation of low-threshold fibers can produce decreases of spontaneous rates below the normal level (e.g., dark blue regions in upper left corner of bottom panel).

In the model, we first created different patterns of ANF deafferentation, ranging from selective deafferentation of high-threshold ANFs to a pattern where all fiber types are affected to a similar degree (see Materials and Methods and Figure 4A for an example), and combined these patterns of deafferentation with different degrees of hearing loss (i.e., increased thresholds; see Materials and Methods and Figure 4B). By this means, we explored a three-dimensional parameter space of damage parameters spanning (i) the degree of deafferentation, (ii) the pattern of deafferentation (i.e., low, medium and high spontaneous ANFs), and (iii) the degree of hearing loss (threshold increase). The model is based on the assumption that in attempting to stabilize neuronal activity following hearing loss, homeostatic plasticity elevates neuronal response gain in the central auditory system, thereby generating increased spontaneous firing rates through over-amplification of spontaneous activity (21, 46, 47, 53, 54). Hyperactivity thus depends first on how much the gain is increased by homeostatic plasticity and, second, on how much spontaneous activity in the AN is reduced through cochlear damage. If the increase in response gain is greater than the reduction of the spontaneous excitatory input from the ANFs, spontaneous hyperactivity develops (46, 47).

Figure 4C shows model results for four different degrees of overall ANF deafferentation from 10 to 40% of the total number of ANFs. For the case where deafferentation affects only high-threshold ANFs (“Ht only”), and thus does not reduce the amount of spontaneous excitatory input provided to the central auditory system by the ANFs, the greater the number of deafferented fibers the higher the spontaneous rate in the central auditory system following homeostasis. However, when low-threshold (and high spontaneous) fibers are also deafferented (i.e., moving from the bottom to the top, “Ht only” to “Equal” in each plot in Figure 4C), the resulting spontaneous firing rates are always lower than when only high-threshold fibers are deafferented. In the “balanced case” where deafferentation affects all fiber types equally (i.e., at the top of each plot in Figure 4C), there is even the suggestion of *hypo*-activity with spontaneous firing rates lower than before cochlear damage (the normal, healthy spontaneous rate is indicated by the dashed line in all plots). Finally, in the model, additional increases of peripheral thresholds always increase spontaneous firing rates in the central auditory system. Therefore, the model demonstrates that the same degree of elevation of spontaneous firing rates can be generated by different types of cochlear damage, and that trajectories exist through the “cochlear damage space” where more damage can even lead to a reduction in spontaneous firing rates in the central auditory nervous system, despite the presence of homeostatic plasticity mechanisms.

### Comparison with Human Data

**Figure 5:**
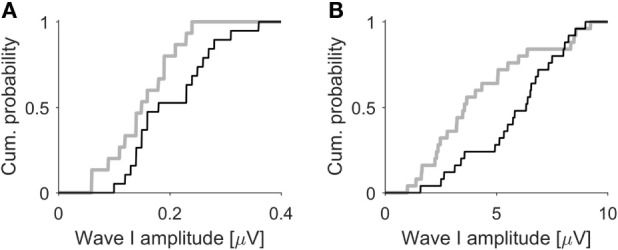
**ABR wave I amplitudes in humans and mice**. **(A)** Cumulative distributions of ABR wave I amplitudes (50 μs clicks, 100 dB SPL) from tinnitus (gray line, *n* = 15) and control participants (black line, *n* = 18), both with normal hearing thresholds. Data from Schaette and McAlpine (21). **(B)** Cumulative distributions of ABR wave I of mice (50 μs clicks, 80 dB SPL, 25 ears from 13 mice) before (black line) and 4 weeks after exposure to octave-band noise at 100 dB SPL (gray line) causing hidden hearing loss. Mice show a similar degree of separation of the ABR amplitude distribution before and after noise-induced hidden hearing loss as do human subjects with and without tinnitus.

The ABR results from the 100-dB exposed mice with HHL resembled our findings from an earlier study in which we compared human tinnitus subjects with clinically normal hearing thresholds to age-, gender-, and hearing-matched control subjects, showing that the tinnitus group had significantly smaller amplitudes of ABR wave I (21). A direct comparison between humans and mice is hampered by the fact that ABR wave I amplitudes in mice are more than a magnitude larger than in humans, due to differences in head size and data acquisition methods. We therefore performed a qualitative comparison of the cumulative distributions of ABR wave I amplitudes. Figure 5A shows the cumulative distribution of the amplitudes of ABR wave I elicited by 50 μs clicks at 100 dB SPL for human tinnitus (gray line, *n* = 15 ears) and control participants (black line, *n* = 18 ears). Despite the significant difference in average amplitude, there is a considerable overlap in the distributions. Figure 5B shows cumulative distributions of ABR wave I amplitudes (50 μs clicks at 80 dB SPL) for mice before (black line) and 4 weeks after (gray line, 25 ears from 13 mice in both cases) exposure to octave-band noise at 100 dB SPL (note that this data set includes additional mice that did not undergo IC recordings). On a qualitative level, there is a great degree of similarity between mice before and after noise-induced HHL and normal-hearing humans with and without tinnitus, suggesting that mice with noise-induced HHL approximate the ABR-phenotype of human tinnitus subjects with normal hearing thresholds and could therefore, if tinnitus is verified through behavioral tests, potentially serve as an animal model for the condition.

## Discussion

We examined peripheral and central auditory function in mice exposed to different levels of noise; 100 dB SPL to elicit temporary shifts in hearing thresholds but long-term deafferentation of IHC synapses, and 105 dB SPL to elicit both permanent threshold shifts and deafferentation. A striking finding is the non-monotonic relationship between the severity of cochlear damage and the degree to which spontaneous firing rates in the ICc were elevated, with less damage leading to more hyperactivity (Figures 2B,C). From previous physiological studies (33, 36), it might have been expected that this relationship would be reversed with higher spontaneous rates following exposure to the more intense sound. Nevertheless, a relatively simple computational model suggests that this seemingly paradoxical outcome might be explained by different degrees of deafferentation of high-spontaneous-rate ANFs (Figure 4C). In particular, the model can account for the data in the frequency range from 12 to 24 kHz, where IC recordings from both exposure groups show similar spontaneous firing rates (Figure 2C) despite significant differences in thresholds following noise exposure (Figures 1A and 2D). The model therefore provides a cogent explanation as to why the permanent hearing threshold shift in mice exposed to the higher intensity (105 dB SPL) noise did not lead to an additional increase in spontaneous firing rates: in these animals, a higher proportion of low-threshold ANFs with high spontaneous rates might have been deafferented. While the exact “trajectory” through the “deafferentation and damage plane” generated by different noise exposures remains to be determined, the modeling results provide a simple framework for understanding the complex relationship between cochlear damage and central auditory hyperactivity.

Our noise exposure paradigm for HHL was modeled on that of Kujawa and Liberman (17), who exposed awake mice to octave-band noise (8–16 kHz) at 100 dB SPL. They reported only temporary shifts in ABR thresholds, but permanently reduced ABR wave I amplitudes at higher sound intensities, as well as a reduced number of synaptic ribbons of IHCs, indicative of deafferentation. Our other noise exposure at 105 dB SPL generated additional, permanent shifts in hearing thresholds and a slightly higher degree of loss of synaptic ribbons in the cochlea, qualitatively similar to the findings of Rüttiger et al., who used noise exposure durations of 1 and 1.5 h at 120 dB SPL, and observed more hearing threshold increase and loss of synaptic ribbons after the longer exposure. In both of our exposure groups, the centrally generated ABR wave IV showed a certain degree of recovery of the amplitudes, a finding also consistent with results of recent studies in mice (55) and rats (56).

We observed significant increases in ICc spontaneous firing rates in both exposure groups. Data from the animals exposed to 100-dB-SPL noise suggest that HHL can lead to the development of increased spontaneous firing rates, an outcome we previously predicted from a computational model designed to account for the effects of deafferentation of high-threshold ANFs (21). In mice with HHL, a prominent peak in the profile of spontaneous firing rates was seen in the CF range of 8–12 kHz, toward the lower end of the octave-band noise used for noise exposure (another, slightly higher peak was found in the CF range of 24–32 kHz, but only four units were recorded there). A similar relationship between noise exposure frequency range, temporary threshold shift, and neuronal hyperactivity has recently been reported for the DCN (57). In this frequency range, noise exposure might only cause a limited degree of ANF deafferentation (17, 58), accentuating the non-linear nature of the relation between cochlear damage and central hyperactivity, which might be influenced by a combination of factors, e.g., central compensation for peripheral damage (46, 54) in conjunction with a loss of lateral inhibition (59–62).

In the animals exposed to noise at 105 dB SPL, we observed the strongest increase in spontaneous firing rates at around 12–16 kHz, where hearing thresholds were permanently increased, matching recent reports from guinea pigs (36) and hamsters (63). It should be noted that previous studies usually only employed a single noise exposure level, either a high-level noise exposure causing permanent hearing loss (36, 41, 42, 49, 63), or a less severe exposure inducing only temporary shifts in hearing thresholds (26, 40, 57, 64), which could explain why the non-monotonic relation between the extent of cochlear damage and neuronal hyperactivity has not been reported before. Also, results indicating a monotonic relation between threshold increase and hyperactivity [e.g., Ref. (36, 44)] have been obtained analyzing different degrees of threshold elevation along the tonotopic axis for a single noise exposure level, and we have observed a similar pattern in the 105 dB SPL group.

An important difference between the two exposure group in our study could be the degree of damage to outer hair cells. In the 105 dB SPL group, which suffered a permanent threshold increase due to the noise exposure, permanent damage to or even loss of outer hair cells might have occurred (65). In the 100 dB SPL exposure group, the absence of a permanent threshold shift suggests that OHCs might have remained largely intact (17, 65). However, to quantify the contribution of OHC damage to our findings, measurements of otoacoustic emissions and detailed hair cell counts would be required. A quantification of hair cell loss would also be an interesting aspect for future studies, as there is also a non-linear relation between cisplatin-induced hair cell loss and central auditory hyperactivity (33): DCN hyperactivity was proportional to the degree of outer hair cell loss for low doses of cisplatin that did not cause IHC loss, whereas additional IHC loss decreased hyperactivity. We have replicated this effect in an earlier modeling study (46) using a simpler version of our current model. These findings indicate that there might be at least one additional point where the relation between noise-induced cochlear damage and hyperactivity is non-monotonic: Noise exposures causing slightly more damage than our 105 dB SPL exposure (e.g., 110 or 115 dB SPL for 2 h) may cause a stronger elevation of spontaneous firing rates, whereas for noise exposures severe enough to cause death of IHCs, another drop in spontaneous firing rates might occur. Such effects could further contribute to the heterogeneity observed in the relation between hearing loss and tinnitus. However, a detailed mapping of the effects of severe noise exposures was beyond the scope of our current study.

Several recent studies (40, 57, 66) and our own results indicate that noise exposure leading to TTS is sufficient to trigger the development of neuronal hyperactivity, but a recent study using has reported that spontaneous firing rates in the guinea pig IC remained normal after bilateral exposure to a loud tone that caused TTS (67). It remains to be determined which factors are responsible for this discrepancy (or heterogeneity). For example, there might be a certain “damage threshold” for the development of hyperactivity after acoustic trauma, i.e., a certain minimum degree of TTS or ANF deafferentation might be required to trigger the development of neuronal hyperactivity. A future study could thus investigate a range of noise exposure severities including even lower intensities than employed in our study to test this hypothesis. Moreover, certain types of sound exposures at relatively low levels that are unlikely to cause cochlear damage could still have an effect on spontaneous neuronal activity in the central auditory system. For example, passive long-term exposure to an “enhanced acoustic environment” consisting of random tone pips at 70–80 dB SPL can have a pronounced effect on spontaneous firing rates in the auditory cortex, with decreases in units with CFs in the frequency range of the tone pips, and increases in units with CFs above and below the exposure frequency range (68, 69). These “side-band” increases have been attributed to plastic changes in gain and lateral inhibition (62). The shape of the profile of spontaneous firing rates that we observed in the mice exposed to noise at 100 dB SPL bears a certain resemblance to the pattern observed for low-level noise, albeit with the crucial difference that we found increases in spontaneous firing also in the frequency range of the noise exposure stimulus (Figures 2B,C). A possible explanation might be an interaction between changes in lateral inhibition and homeostatic compensation for decreased input from the auditory nerve. This scenario could be investigated in more detail in future studies. Another interesting finding of the low-level noise exposure studies was changes in the tonotopic organization of the auditory cortex (68, 70) and the auditory midbrain (71) through exposure to tone pip environments or broadband noise for weeks at levels of 70–80 dB SPL. Such reorganization processes could also explain why we encountered relatively more units with CFs in the range of 8–12 kHz in the noise-exposed groups compared to the control group (Figure 2A), but it remains to be determined if a relatively short duration, as the 2 h used in our study, is sufficient to trigger this process in order to determine whether reorganization is driven by the exposure itself, or a consequence of cochlear damage.

Our data do not enable us to determine whether the increase in spontaneous activity in the ICc is generated locally, or inherited from another processing stage in the auditory pathway. It has recently been demonstrated that ablation of the contralateral DCN abolishes noise-induced hyperactivity in the IC (63), suggesting that neuronal hyperactivity in the IC could be generated through propagation of elevated spontaneous activity from the DCN. A potential mechanism might be amplification of spontaneous excitatory input from the auditory nerve, as silencing the auditory nerve 4 weeks after noise trauma through cooling or application of TTX abolishes hyperactivity in the IC (36). Beyond 4 weeks, however, spontaneous neuronal hyperactivity appears to become a persistent feature in the central auditory system, as silencing spontaneous auditory nerve activity no longer reduces central hyperactivity (37). In the DCN, neuronal hyperactivity has been linked to a reduction of neural inhibition (26), and altered auditory-somatosensory integration might also play a role (72, 73). In the IC of normal-hearing animals, blocking inhibition through iontophoretic application of the GABA antagonist bicuculline leads to a substantial increase in spontaneous activity (74), suggesting a potential mechanism for the generation of hyperactivity within the IC.

An open question is whether the mice in our study were experiencing tinnitus – we did not perform behavioral tests. Several recent studies have reported behavioral evidence of tinnitus in mice (26, 66, 75), rats (40), gerbils (76), and guinea pigs (34, 57) following noise exposure leading to temporary shifts in hearing thresholds. Tinnitus-like behavior was reported for the majority, but not all of the animals (26, 34, 40). Similarly, several previous studies have demonstrated that noise exposure leading to permanently elevated hearing thresholds can also generate behavioral signs of tinnitus (41–43, 56, 77). Therefore, some proportion of the mice in our study with “hidden” as well as “normal” hearing loss might be expected to have had tinnitus. Behavioral verification of tinnitus would also help to clarify whether mice with HHL might be a suitable model for studying tinnitus with normal hearing, as suggested by the qualitative similarity between the ABR phenotype of our mice with HHL and human tinnitus subjects with normal hearing (Figure 5). Moreover, it has recently also been suggested that mice might show signs of hyperacusis rather than tinnitus after exposure to octave-band noise at 100 dB SPL (55), emphasizing the importance of behavioral testing for future studies to disentangle the effects of different auditory pathologies.

Could the increased spontaneous firing rates we observed in the IC of noise-exposed mice represent a neural correlate of tinnitus? The relationship between exposure frequency range and the location of increases in spontaneous activity on the tonotopic axis of the IC in our study is similar to the relation between exposure frequency range and the putative tinnitus pitch in gerbils (76). Moreover, in both the cochlear nucleus (34) and the inferior colliculus (43), a close link between spontaneous neuronal hyperactivity and behavioral evidence for tinnitus has been reported. Interestingly, it has recently been demonstrated that acoustic trauma that induces only temporary hearing threshold shifts might actually be more likely to induce tinnitus than a noise exposure causing permanent threshold elevation (78), an observation that is closely matched by our finding that the “HHL” noise exposure evoked a greater elevation of spontaneous firing rates in the IC than the more severe noise exposure. However, neuronal hyperactivity may not always be tinnitus-specific; in a recent study in guinea pigs, increases in spontaneous firing rates were observed in all noise-exposed animals, regardless of whether they showed behavioral signs of tinnitus or not (79).

One important difference between our study and most other studies on animal models of tinnitus is that we employed bilateral, rather than unilateral, noise exposure. Although unilateral noise exposure has the advantage of preserving hearing in one ear, which can then serve as a within-subject control (and ensures normal hearing abilities for behavioral testing in at least one ear), noise exposure bilaterally is more representative of the case in human listeners, where usually both ears are exposed to noise (with rifle shooting being possibly the most prominent exception). It remains to be determined whether bilateral noise exposure is more or less likely to lead to tinnitus in animals. We have recently demonstrated that unilateral earplugging can lead to the perception of phantom sounds in normal-hearing human volunteers (51), which is qualitatively similar to the reports of tinnitus during bilateral auditory deprivation in an anechoic chamber (80, 81). Further, the degree of elevation of spontaneous activity in the inferior colliculus we observed following bilateral noise exposure appears similar to the effects reported for unilateral noise exposure (36, 38, 79, 82).

An important insight from our electrophysiological and modeling results is that they offer an indication of why hearing loss might not always lead to tinnitus. If the relationship between cochlear damage and the strength of a putative neuronal correlate for tinnitus is indeed non-monotonic, then some specific patterns or configurations of cochlear damage might be more likely to lead to tinnitus than others. This finding could offer an interesting new approach toward understanding the pathophysiology of tinnitus and tinnitus heterogeneity, and for evaluating potential tinnitus triggers.

## Ethics

The human data presented in this manuscript is from a previous study (21), which had been approved by the University College London Research Ethics Committee.

## Author Contributions

LH designed research, performed experiments, analysed data, and wrote the manuscript. WB performed experiments and wrote the manuscript. LA performed experiments. H-CO performed experiments and analysed data. JA supervised experiments, analysed data, and wrote the manuscript. DM designed research, supervised experiments, and wrote the manuscript. JL designed research, supervised experiments, and wrote the manuscript. RS designed research, analysed data, produced all figures, and wrote the manuscript.

## Conflict of Interest Statement

The authors declare that the research was conducted in the absence of any commercial or financial relationships that could be construed as a potential conflict of interest.

## References

[1] Axelsson A Ringdahl A Tinnitus – a study of its prevalence and characteristics. Br J Audiol (1989) 23:53–62. 10.3109/03005368909077819 2784987

[2] Nicolas-Puel C Faulconbridge RL Guitton M Puel JL Mondain M Uziel A. Characteristics of tinnitus and etiology of associated hearing loss: a study of 123 patients. Int Tinnitus J (2002) 8:37–44. 14763234

[3] Chung DY Gannon RP Mason K. Factors affecting the prevalence of tinnitus. Audiology (1984) 23:441–52. 10.3109/00206098409070084 6487142

[4] Ayache D Earally F Elbaz P. Characteristics and postoperative course of tinnitus in otosclerosis. Otol Neurotol (2003) 24:48–51. 10.1097/00129492-200301000-00011 12544028

[5] Sobrinho PG Oliveira CA Venosa AR. Long-term follow-up of tinnitus in patients with otosclerosis after stapes surgery. Int Tinnitus J (2004) 10:197–201. 15732523

[6] Nosrati-Zarenoe R Arlinger S Hultcrantz E. Idiopathic sudden sensorineural hearing loss: results drawn from the Swedish national database. Acta Otolaryngol (2007) 127:1168–75. 10.1080/00016480701242477 17851927

[7] Henry JA Meikle M Gilbert A Audiometric correlates of tinnitus pitch: Insights from the Tinnitus Data Registry. In: Hazell J, editor. VI. International Tinnitus Seminar. London: The Tinnitus and Hyperacusis Centre (1999). p. 51–7.

[8] König O Schaette R Kempter R Gross M. Course of hearing loss and occurrence of tinnitus. Hear Res (2006) 221:59–64. 10.1016/j.heares.2006.07.007 16962270

[9] Pan T Tyler RS Ji H Coelho C Gehringer AK Gogel SA. The relationship between tinnitus pitch and the audiogram. Int J Audiol (2009) 48:277–94. 10.1080/14992020802581974 19842803

[10] Sereda M Hall DA Bosnyak DJ Edmondson-Jones M Roberts LE Adjamian P Re-examining the relationship between audiometric profile and tinnitus pitch. Int J Audiol (2011) 50:303–12. 10.3109/14992027.2010.551221 21388238 PMC3082165

[11] Barnea G Attias J Gold S Shahar A. Tinnitus with normal hearing sensitivity: extended high-frequency audiometry and auditory-nerve brain-stem-evoked responses. Audiology (1990) 29:36–45. 10.3109/00206099009081644 2310352

[12] Sanchez TG Medeiros IR Levy CP Ramalho Jda R Bento RF. Tinnitus in normally hearing patients: clinical aspects and repercussions. Braz J Otorhinolaryngol (2005) 71:427–31. 10.1016/S1808-8694(15)31194-0 16446955 PMC9441966

[13] Lockwood AH Salvi RJ Burkard RF Tinnitus. N Engl J Med (2002) 347:904–10. 10.1056/NEJMra013395 12239260

[14] Davis B Qiu W Hamernik RP. The use of distortion product otoacoustic emissions in the estimation of hearing and sensory cell loss in noise-damaged cochleas. Hear Res (2004) 187:12–24. 10.1016/S0378-5955(03)00339-3 14698083

[15] Moore BC Huss M Vickers DA Glasberg BR Alcantara JI. A test for the diagnosis of dead regions in the cochlea. Br J Audiol (2000) 34:205–24. 10.3109/03005364000000131 10997450

[16] Summers V Makashay MJ Theodoroff SM Leek MR. Suprathreshold auditory processing and speech perception in noise: hearing-impaired and normal-hearing listeners. J Am Acad Audiol (2013) 24:274–92. 10.3766/jaaa.24.4.4 23636209

[17] Kujawa SG Liberman MC. Adding insult to injury: cochlear nerve degeneration after “temporary” noise-induced hearing loss. J Neurosci (2009) 29:14077–85. 10.1523/JNEUROSCI.2845-09.2009 19906956 PMC2812055

[18] Furman AC Kujawa SG Liberman MC. Noise-induced cochlear neuropathy is selective for fibers with low spontaneous rates. J Neurophysiol (2013) 110:577–86. 10.1152/jn.00164.2013 23596328 PMC3742994

[19] Makary CA Shin J Kujawa SG Liberman MC Merchant SN. Age-related primary cochlear neuronal degeneration in human temporal bones. J Assoc Res Otolaryngol (2011) 12:711–7. 10.1007/s10162-011-0283-2 21748533 PMC3214241

[20] Sergeyenko Y Lall K Liberman MC Kujawa SG. Age-related cochlear synaptopathy: an early-onset contributor to auditory functional decline. J Neurosci (2013) 33:13686–94. 10.1523/JNEUROSCI.1783-13.2013 23966690 PMC3755715

[21] Schaette R McAlpine D. Tinnitus with a normal audiogram: physiological evidence for hidden hearing loss and computational model. J Neurosci (2011) 31:13452–7. 10.1523/JNEUROSCI.2156-11.2011 21940438 PMC6623281

[22] Gu JW Herrmann BS Levine RA Melcher JR. Brainstem auditory evoked potentials suggest a role for the ventral cochlear nucleus in tinnitus. J Assoc Res Otolaryngol (2012) 13(6):819–33. 10.1007/s10162-012-0344-1 22869301 PMC3505586

[23] Vale C Sanes DH. The effect of bilateral deafness on excitatory and inhibitory synaptic strength in the inferior colliculus. Eur J Neurosci (2002) 16:2394–404. 10.1046/j.1460-9568.2002.02302.x 12492434

[24] Caspary DM Schatteman TA Hughes LF. Age-related changes in the inhibitory response properties of dorsal cochlear nucleus output neurons: role of inhibitory inputs. J Neurosci (2005) 25:10952–9. 10.1523/JNEUROSCI.2451-05.2005 16306408 PMC6725883

[25] Whiting B Moiseff A Rubio ME. Cochlear nucleus neurons redistribute synaptic AMPA and glycine receptors in response to monaural conductive hearing loss. Neuroscience (2009) 163:1264–76. 10.1016/j.neuroscience.2009.07.049 19646510 PMC2760652

[26] Middleton JW Kiritani T Pedersen C Turner JG Shepherd GM Tzounopoulos T. Mice with behavioral evidence of tinnitus exhibit dorsal cochlear nucleus hyperactivity because of decreased GABAergic inhibition. Proc Natl Acad Sci U S A (2011) 108:7601–6. 10.1073/pnas.1100223108 21502491 PMC3088638

[27] Wang H Yin G Rogers K Miralles C De Blas AL Rubio ME Monaural conductive hearing loss alters the expression of the GluA3 AMPA and glycine receptor alpha1 subunits in bushy and fusiform cells of the cochlear nucleus. Neuroscience (2011) 199:438–51. 10.1016/j.neuroscience.2011.10.021 22044924 PMC3237936

[28] Kotak VC Fujisawa S Lee FA Karthikeyan O Aoki C Sanes DH. Hearing loss raises excitability in the auditory cortex. J Neurosci (2005) 25:3908–18. 10.1523/JNEUROSCI.5169-04.2005 15829643 PMC1764814

[29] Dong S Mulders WH Rodger J Woo S Robertson D. Acoustic trauma evokes hyperactivity and changes in gene expression in guinea-pig auditory brainstem. Eur J Neurosci (2010) 31:1616–28. 10.1111/j.1460-9568.2010.07183.x 20525074

[30] Liberman MC Dodds LW. Single-neuron labeling and chronic cochlear pathology. II. Stereocilia damage and alterations of spontaneous discharge rates. Hear Res (1984) 16:43–53. 10.1016/0378-5955(84)90024-8 6511672

[31] Dallos P Harris D. Properties of auditory nerve responses in absence of outer hair cells. J Neurophysiol (1978) 41:365–83. 650272 10.1152/jn.1978.41.2.365

[32] Kaltenbach JA Godfrey DA Neumann JB McCaslin DL Afman CE Zhang J. Changes in spontaneous neural activity in the dorsal cochlear nucleus following exposure to intense sound: relation to threshold shift. Hear Res (1998) 124:78–84. 10.1016/S0378-5955(98)00119-1 9822904

[33] Kaltenbach JA Rachel JD Mathog TA Zhang J Falzarano PR Lewandowski M. Cisplatin-induced hyperactivity in the dorsal cochlear nucleus and its relation to outer hair cell loss: relevance to tinnitus. J Neurophysiol (2002) 88:699–714. 12163523 10.1152/jn.2002.88.2.699

[34] Koehler SD Shore SE. Stimulus timing-dependent plasticity in dorsal cochlear nucleus is altered in tinnitus. J Neurosci (2013) 33:19647–56. 10.1523/JNEUROSCI.2788-13.2013 24336728 PMC3858633

[35] Vogler DP Robertson D Mulders WH. Hyperactivity in the ventral cochlear nucleus after cochlear trauma. J Neurosci (2011) 31:6639–45. 10.1523/JNEUROSCI.6538-10.2011 21543592 PMC6632868

[36] Mulders WH Robertson D. Hyperactivity in the auditory midbrain after acoustic trauma: dependence on cochlear activity. Neuroscience (2009) 164:733–46. 10.1016/j.neuroscience.2009.08.036 19699277

[37] Mulders WH Robertson D. Progressive centralization of midbrain hyperactivity after acoustic trauma. Neuroscience (2011) 192:753–60. 10.1016/j.neuroscience.2011.06.046 21723924

[38] Vogler DP Robertson D Mulders WH. Hyperactivity following unilateral hearing loss in characterized cells in the inferior colliculus. Neuroscience (2014) 265:28–36. 10.1016/j.neuroscience.2014.01.017 24468107

[39] Norena AJ Eggermont JJ. Changes in spontaneous neural activity immediately after an acoustic trauma: implications for neural correlates of tinnitus. Hear Res (2003) 183:137–53. 10.1016/S0378-5955(03)00225-9 13679145

[40] Engineer ND Riley JR Seale JD Vrana WA Shetake JA Sudanagunta SP Reversing pathological neural activity using targeted plasticity. Nature (2011) 470:101–4. 10.1038/nature09656 21228773 PMC3295231

[41] Kaltenbach JA Zacharek MA Zhang J Frederick S. Activity in the dorsal cochlear nucleus of hamsters previously tested for tinnitus following intense tone exposure. Neurosci Lett (2004) 355:121–5. 10.1016/j.neulet.2003.10.038 14729250

[42] Ahlf S Tziridis K Korn S Strohmeyer I Schulze H. Predisposition for and prevention of subjective tinnitus development. PLoS One (2012) 7:e44519. 10.1371/journal.pone.0044519 23056180 PMC3462765

[43] Mulders WH Barry KM Robertson D. Effects of furosemide on cochlear neural activity, central hyperactivity and behavioural tinnitus after cochlear trauma in Guinea pig. PLoS One (2014) 9:e97948. 10.1371/journal.pone.0097948 24835470 PMC4023991

[44] Mulders WH Ding D Salvi R Robertson D. Relationship between auditory thresholds, central spontaneous activity, and hair cell loss after acoustic trauma. J Comp Neurol (2011) 519:2637–47. 10.1002/cne.22644 21491427 PMC3140598

[45] Sahani M Latent Variable Models for Neural Data Analysis. Pasadena, CA: California Institute of Technology (1999).

[46] Schaette R Kempter R. Development of tinnitus-related neuronal hyperactivity through homeostatic plasticity after hearing loss: a computational model. Eur J Neurosci (2006) 23:3124–38. 10.1111/j.1460-9568.2006.04774.x 16820003

[47] Schaette R Kempter R. Development of hyperactivity after hearing loss in a computational model of the dorsal cochlear nucleus depends on neuron response type. Hear Res (2008) 240:57–72. 10.1016/j.heares.2008.02.006 18396381

[48] Young ED Davis KA Circuitry and function of the dorsal cochlear nucleus. In: Oertel D Fay RR Popper AN, editors. Integrative Functions in the Mammalian Auditory Pathway. New York: Springer (2002). p. 121–57.

[49] Manzoor NF Gao Y Licari F Kaltenbach JA Comparison and contrast of noise-induced hyperactivity in the dorsal cochlear nucleus and inferior colliculus. Hear Res (2013) 295:114–23. 10.1016/j.heares.2012.04.003 22521905 PMC3538909

[50] Bourien J Tang Y Batrel C Huet A Lenoir M Ladrech S Contribution of auditory nerve fibers to compound action potential of the auditory nerve. J Neurophysiol (2014) 112:1025–39. 10.1152/jn.00738.2013 24848461

[51] Schaette R Turtle C Munro KJ. Reversible induction of phantom auditory sensations through simulated unilateral hearing loss. PLoS One (2012) 7(6):e35238. 10.1371/journal.pone.0035238 22675466 PMC3366980

[52] Schaette R Computational modeling of tinnitus development. J Acoust Soc Am (2013) 133:3560–3560. 10.1121/1.4806483

[53] Schaette R Kempter R. Predicting tinnitus pitch from patients’ audiograms with a computational model for the development of neuronal hyperactivity. J Neurophysiol (2009) 101:3042–52. 10.1152/jn.91256.2008 19357344

[54] Norena AJ. An integrative model of tinnitus based on a central gain controlling neural sensitivity. Neurosci Biobehav Rev (2011) 35:1089–109. 10.1016/j.neubiorev.2010.11.003 21094182

[55] Hickox AE Liberman MC. Is noise-induced cochlear neuropathy key to the generation of hyperacusis or tinnitus? J Neurophysiol (2014) 111:552–64. 10.1152/jn.00184.2013 24198321 PMC3921399

[56] Rüttiger L Singer W Panford-Walsh R Matsumoto M Lee SC Zuccotti A The reduced cochlear output and the failure to adapt the central auditory response causes tinnitus in noise exposed rats. PLoS One (2013) 8:e57247. 10.1371/journal.pone.0057247 23516401 PMC3596376

[57] Dehmel S Pradhan S Koehler S Bledsoe S Shore S Noise overexposure alters long-term somatosensory-auditory processing in the dorsal cochlear nucleus – possible basis for tinnitus-related hyperactivity? J Neurosci (2012) 32:1660–71. 10.1523/JNEUROSCI.4608-11.2012 22302808 PMC3567464

[58] Liberman LD Suzuki J Liberman MC. Dynamics of cochlear synaptopathy after acoustic overexposure. J Assoc Res Otolaryngol (2015) 16:205–19. 10.1007/s10162-015-0510-3 25676132 PMC4368657

[59] Gerken GM. Central tinnitus and lateral inhibition: an auditory brainstem model. Hear Res (1996) 97:75–83. 10.1016/S0378-5955(96)80009-8 8844188

[60] Kral A Majernik V. On lateral inhibition in the auditory system. Gen Physiol Biophys (1996) 15:109–27. 8899416

[61] Parra LC Pearlmutter BA. Illusory percepts from auditory adaptation. J Acoust Soc Am (2007) 121:1632–41. 10.1121/1.2431346 17407900

[62] Pienkowski M Eggermont JJ. Reversible long-term changes in auditory processing in mature auditory cortex in the absence of hearing loss induced by passive, moderate-level sound exposure. Ear Hear (2012) 33:305–14. 10.1097/AUD.0b013e318241e880 22343545

[63] Manzoor N Licari FG Klapchar M Elkin R Gao Y Kaltenbach JA Noise-induced hyperactivity in the inferior colliculus: its relationship with hyperactivity in the dorsal cochlear nucleus. J Neurophysiol (2012) 108:976–88. 10.1152/jn.00833.2011 22552192 PMC3424082

[64] Brozoski TJ Bauer CA Caspary DM. Elevated fusiform cell activity in the dorsal cochlear nucleus of chinchillas with psychophysical evidence of tinnitus. J Neurosci (2002) 22:2383–90. 11896177 10.1523/JNEUROSCI.22-06-02383.2002 PMC6758251

[65] Chen GD Fechter LD. The relationship between noise-induced hearing loss and hair cell loss in rats. Hear Res (2003) 177:81–90. 10.1016/S0378-5955(02)00802-X 12618320

[66] Longenecker RJ Galazyuk AV. Development of tinnitus in CBA/CaJ mice following sound exposure. J Assoc Res Otolaryngol (2011) 12:647–58. 10.1007/s10162-011-0276-1 21667173 PMC3173549

[67] Heeringa AN van Dijk P. The dissimilar time course of temporary threshold shifts and reduction of inhibition in the inferior colliculus following intense sound exposure. Hear Res (2014) 312:38–47. 10.1016/j.heares.2014.03.004 24650953

[68] Norena AJ Gourevitch B Aizawa N Eggermont JJ. Spectrally enhanced acoustic environment disrupts frequency representation in cat auditory cortex. Nat Neurosci (2006) 9:932–9. 10.1038/nn0906-1193a 16783369

[69] Munguia R Pienkowski M Eggermont JJ. Spontaneous firing rate changes in cat primary auditory cortex following long-term exposure to non-traumatic noise: tinnitus without hearing loss? Neurosci Lett (2013) 546:46–50. 10.1016/j.neulet.2013.04.048 23648387

[70] Pienkowski M Eggermont JJ. Long-term, partially-reversible reorganization of frequency tuning in mature cat primary auditory cortex can be induced by passive exposure to moderate-level sounds. Hear Res (2009) 257:24–40. 10.1016/j.heares.2009.07.011 19647789

[71] Lau C Pienkowski M Zhang JW McPherson B Wu EX. Chronic exposure to broadband noise at moderate sound pressure levels spatially shifts tone-evoked responses in the rat auditory midbrain. Neuroimage (2015) 122:44–51. 10.1016/j.neuroimage.2015.07.065 26232718

[72] Zeng C Nannapaneni N Zhou J Hughes LF Shore S. Cochlear damage changes the distribution of vesicular glutamate transporters associated with auditory and nonauditory inputs to the cochlear nucleus. J Neurosci (2009) 29:4210–7. 10.1523/JNEUROSCI.0208-09.2009 19339615 PMC4487620

[73] Zeng C Yang Z Shreve L Bledsoe S Shore S. Somatosensory projections to cochlear nucleus are upregulated after unilateral deafness. J Neurosci (2012) 32:15791–801. 10.1523/JNEUROSCI.2598-12.2012 23136418 PMC3501653

[74] McAlpine D Palmer AR. Blocking GABAergic inhibition increases sensitivity to sound motion cues in the inferior colliculus. J Neurosci (2002) 22:1443–53. 11850471 10.1523/JNEUROSCI.22-04-01443.2002 PMC6757567

[75] Turner J Larsen D Hughes L Moechars D Shore S. Time course of tinnitus development following noise exposure in mice. J Neurosci Res (2012) 90:1480–8. 10.1002/jnr.22827 22434653 PMC3725635

[76] Nowotny M Remus M Kossl M Gaese BH. Characterization of the perceived sound of trauma-induced tinnitus in gerbils. J Acoust Soc Am (2011) 130:2827–34. 10.1121/1.3646902 22087911

[77] Heffner HE Heffner RS Behavioural tests for tinnitus in animals. In: Eggermont JJ Zeng FG, editors. Tinnitus (Springer Handbook of Auditory Research). New York, Heidelberg, Dordrecht, London: Springer (2012). p. 21–58.

[78] Kiefer L Schauen A Abendroth S Gaese BH Nowotny M. Variation in acoustic overstimulation changes tinnitus characteristics. Neuroscience (2015) 310:176–87. 10.1016/j.neuroscience.2015.09.023 26365609

[79] Coomber B Berger JI Kowalkowski VL Shackleton TM Palmer AR Wallace MN. Neural changes accompanying tinnitus following unilateral acoustic trauma in the guinea pig. Eur J Neurosci (2014) 40:2427–41. 10.1111/ejn.12580 24702651 PMC4215599

[80] Heller MF Bergman M Tinnitus aurium in normally hearing persons. Ann Otol Rhinol Laryngol (1953) 62:73–83. 10.1177/000348945306200107 13041055

[81] Del Bo L Forti S Ambrosetti U Costanzo S Mauro D Ugazio G Tinnitus aurium in persons with normal hearing: 55 years later. Otolaryngol Head Neck Surg (2008) 139:391–4. 10.1016/j.otohns.2008.06.019 18722219

[82] Mulders WH Robertson D. Development of hyperactivity after acoustic trauma in the guinea pig inferior colliculus. Hear Res (2013) 298:104–8. 10.1016/j.heares.2012.12.008 23276730

